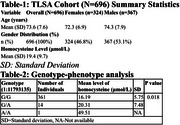# Comprehensive Analysis of MTHFR Polymorphisms in the TATA Longitudinal Study of Aging Cohort

**DOI:** 10.1002/alz70855_106491

**Published:** 2025-12-24

**Authors:** Sohan Angelo A, Piu A Das, Abhishek Mensegere Lingegodwa, Krithika Subramaniam, Khader Valli Rupanagudi, Albert Stezin, Amitha A CM, Gauri Mullerpattan, Vidhya R, Jonas S Sundarakumar, Thomas Gregor Issac, Prathima Arvind

**Affiliations:** ^1^ Center for Brain Research, Bangalore Urban, Karnataka, India; ^2^ Centre for Brain Research, Indian Institute of Science, Bangalore, Karnataka, India; ^3^ Centre for Brain Research, Indian Institute of Science, Bangalore, India; ^4^ Center for Brain Research, Bangalore, India; ^5^ Center for Brain Research, Bangalore Urban, India

## Abstract

**Background:**

Methylenetetrahydrofolate reductase (MTHFR) is an essential enzyme for folate metabolism and homocysteine regulation. Genetic variants in MTHFR have been associated with elevated homocysteine levels, which are linked to various health issues, including cognitive decline (1,2). The current study investigated the genetic variations in the MTHFR gene and its flanking regions (1mb upstream and 1mb downstream) and their association with homocysteine level in the TATA Longitudinal Study of Aging (TLSA) cohort.

**Method:**

The study involved *N* = 696 TLSA participants. Mean age and homocysteine level were calculated using Mann‐Whitney and gender proportion by Chi‐Squared. QualityControl(QC) and phenotype‐genotype analysis were carried using PLINKv1.9.0. After QC analysis a total of 376 participants were considered for genotype‐phenotype analysis.

**Result:**

The cohort had a mean age of 73.6 years (SD = 7.6), with 46.8% being female. Additionally, the TLSA cohort exhibited elevated homocysteine levels, with a mean of 19.4 μmol/L, significantly exceeding the normal range of 5‐15 μmol/L (Table‐1). In The intronic variant 1:11793135 in the MTHFR gene, with an allele frequency of 0.0213, shows a significant association with homocysteine levels (*p* = 0.01867, adjusted). The analysis was conducted using PLINK, accounting for age, gender, vitamin B12, and folic acid levels.

**Conclusion:**

This study provides important new insights into the genetic architecture of MTHFR variants in an elderly Indian population, paving the way for further research aimed at mitigating cognitive decline associated with aging. The identification of a significant association between the intronic variant 1:11793135 and elevated homocysteine levels suggests a potential genetic predisposition to altered homocysteine metabolism in aging individuals. These findings emphasize the importance of genetic screening for MTHFR polymorphisms in at‐risk populations and highlight the need for targeted nutritional and pharmacological interventions to regulate homocysteine levels.